# READMISSION RISK BY INSURANCE TYPE FOR PATIENTS WITH HEART FAILURE IN SKILLED NURSING FACILITIES

**DOI:** 10.1093/geroni/igz038.2395

**Published:** 2019-11-08

**Authors:** Andrea E Daddato, Edward A Miller, Pamela Nadash, Denise Tyler, Rebecca S Boxer

**Affiliations:** 1 University of Colorado School of Medicine, Aurora, Colorado, United States; 2 University of Massachusetts Boston, Boston, Massachusetts, United States; 3 RTI International, Waltham, Massachusetts, United States; 4 Kaiser Permanente Institute for Health Research, Aurora, Colorado, United States

## Abstract

Heart failure (HF) is a leading cause of potentially preventable hospital readmissions for Medicare beneficiaries from skilled nursing facilities (SNFs). This research seeks to determine if a HF patient’s insurance type (Medicare Fee-for-Service (FFS) vs. Medicare Advantage (MA)) influences their risk for readmission within 30 days of hospital discharge to a SNF. MA beneficiaries receive benefits through managed care plans with restricted networks, but typically expanded benefits. This research is particularly timely in light of CMS’ new penalties under the Protecting Access to Medicare Act (PAMA) directed at SNFs for 30-day rehospitalizations. Outcomes data on readmissions from a randomized controlled trial of HF Disease Management in SNFs conducted from 2014-2017 were used to evaluate the risk of readmission. Patients with HF receiving SNF care were enrolled and followed for 30 days from SNF admission. Patients were recruited from 29 primarily for-profit (93%) SNFs that contracted with an average of 4.07 (±5.48) MA plans. Of the 340 study participants followed, 62% had FFS Medicare coverage (n=212) and 38% had MA (n=128). In total, 23% (n=79) of patients experienced at least one readmission within 30 days of hospital discharge. FFS patients had a higher risk of rehospitalization within 30 days of hospital discharge than MA patients (25% vs. 20%), but the association between insurance type and rehospitalization was not statistically significant (p-value=0.177). Findings suggest that insurance type may be an important risk factor for rehospitalizations for patients with HF from SNF; however, a larger sample will need to confirm this relationship.

